# Digital Contact Tracing and COVID-19: Design, Deployment, and Current Use in Italy

**DOI:** 10.3390/healthcare10010067

**Published:** 2021-12-30

**Authors:** Noemi Scrivano, Rosario Alfio Gulino, Daniele Giansanti

**Affiliations:** 1 Facoltà di Ingegneria, Università di Tor Vergata, 00133 Roma, Italy; noemi-scrivano@hotmail.com (N.S.); rosario.gulino.uni.tv@hotmail.com (R.A.G.); 2 Centro Tisp, Istituto Superiore di Sanità, 00161 Roma, Italy

**Keywords:** eHealth, medical devices, digital health, mHealth, cyber-risk, contact tracing, digital health, app, pandemic, COVID-19

## Abstract

The technological innovation of digital contact tracing (DCT) has certainly characterized the COVID-19 pandemic, as compared to the previous ones. Based on the first studies, considerable support was expected from smartphone applications (“apps”) for DCT. This commentary focuses on digital contact tracing. Its contributions are threefold: (a) Recall the initial expectations of these technologies and the state of diffusion. (b) Deal with the introduction of the app “Immuni” in Italy, while also highlighting the initiatives undertaken at the government level. (c) Report the state of diffusion and use of this App. The commentary ends by proposing some reflections on the continuation of this investigation in Italy.

## 1. Introduction

In the *health domain*, contact tracing (CT) is defined by the World Health Organization [1] to be composed of three activities:

(a) Contact identification,
(b) Contact listing, and
(c) Contact follow-up.

In this pandemic, unlike the previous ones, we have been able to rely on strong technological innovation in mobile technology as we know it today, which is based on smartphones (available in their current configuration starting from 2007 [2]). Immediately at the beginning of the pandemic, the potential of mobile technology as a strategic support tool for controlling the spread of the pandemic, emerged through modeling studies. Ferretti et al. [3] demonstrated that the use of digital contact tracing (DCT) [3] could control the diffusion of the COVID-19 (transforming the three components of the CT into the three components of the DCT). Indeed, in some cases, DCT seems irreplaceable. Just think of super diffusion events, or when it is impossible for a person to remember all the recent contacts.

Subsequently, DCT has been considered as a powerful and strategic tool capable of transforming the traditional CT with a practical, effective, speedy, and reliable digital approach. Solutions with a different technological approach have been developed quickly in the first few months of the pandemic. Apps were deployed using GPS or Bluetooth (with different technological variants) for DCT, with different approaches to privacy [4]. DCT also used other solutions, such as in China [5]. A national app was not developed here. WeChat and Alipay were used in China to convey a security code (*Healthcode*) for DCT. In the following months, the use of DCT has spread, and, to date, there is consolidated scientific literature on this experience of using technology in the *health domain*.

The purpose of the commentary is: (*a*) to recall the state of diffusion of DCT to date. *(b)* To highlight the initiatives undertaken at the government level for the *running in* of the Italian DCT based on the App, “Immuni”. (*c*) To report the state of diffusion and use. The remainder of this commentary is arranged in three sections, followed by concluding perspectives.

Section 2 (The digital contact tracing: the state of diffusion of the technology) takes stock of the diffusion of technology in the health domain. Section 3 (The Italian national app, “Immuni”, for digital contact tracing: the running-in and the initiatives supporting the diffusion) deals with the introduction of the App, “Immuni”, and the government initiatives undertaken in Italy. Section 4 (State of diffusion and use of the app, “Immuni”) reports and discusses the state of diffusion and use of DCT in Italy.

## 2. The Digital Contact Tracing: Design, Deployment, and Current Use

A search on Pubmed (as of 5 October 2021) with the key *((Contact tracing [Title/Abstract]) AND (App))* returned 176 results, of which 172 (97.73%) were published between 2020–2021. Before the pandemic DCT had been used in the field of tuberculosis [6] and hepatitis [7]. Among these articles, 21 are reviews or overviews, as they were found by the search terms *((Contact tracing [Title/Abstract]) AND (App)) AND (review)*, 20 of which were released from the last two years. A total of 13 reviews and overviews are very recent, as they appeared in 2021. They deal with heterogeneous aspects of DCT development. They concern *census, privacy, functionality, integrations with other systems, integration acceptance, quality, effectiveness, and other issues*.

To date, more than 78 countries have developed COVID-19 DCT apps to limit the spread of the coronavirus [8]. An analysis of the literature shows that Bluetooth is one of the major technologies used in DCT [9]. Europe, for example, proposed at least two digital contact tracing application models, one described based on privacy-preserving proximity tracing [10] with calculations on the mobile phone, and the other based on pan-European privacy-preserving proximity tracing [11], with calculations on a central server. The approach relating to the collection of information (to be entered into the system) was different between the different apps. For example, The Norwegian, Singaporean, Georgian, and New Zealand apps were among those that collected the most personal information from users, whereas some apps, such as the Swiss app and the Italian (“Immuni”) app, did not collect any user information [9].

The study proposed in [12] reviewed the functionalities and effectiveness of the free mobile health applications available in the Google Play and App stores in some nations during the COVID-19 outbreak [12]. The analysis revealed that various applications have been developed for different functions, such as contact tracing, awareness building, appointment booking, online consultation, etc. However, the study highlighted that only a few applications have integrated various functions and features (e.g., self-assessment, consultation, support, and access to information). No apps were identified that had built-in social media features. Very few apps were dedicated to raising awareness and sharing information about the COVID-19 pandemic. The study [12] suggested developing integrated mobile health applications with most of the features, including DCT. The study reported in [13] considered the quality of the apps for DCT. It used the mobile app rating scale to assess the app quality. It highlighted that European national health authorities have generally released high quality COVID-19 contact tracing apps, about functionality, aesthetics, and information quality. However, the study reported that the engagement-oriented design generally was of lower quality. A lot of both *technological and medical knowledge* has been collected. There are now studies, such as [14], which derive and summarize best practices for the design of the ideal digital contact tracing apps.

## 3. The Italian National App, “Immuni”, for Digital Contact Tracing: The *Running-In* and the Initiatives Supporting the Diffusion

Italy released its own national app called “*Immuni*”. The use (download and data entry) is on a voluntary basis [15].

Italian politicians have opted for a centralized and non-regionalized approach for the use of an app for DCT. A government app was therefore developed after an appropriate public selection of various proposals [16]. Updated information and project data, with a high-level description, are available in [15,17]. In brief, this app uses Bluetooth low energy technology to distinguish proximity events between citizens using a smartphone with the app installed.

The introduction of the app, “Immuni”, was accompanied by dissemination initiatives for all the actors involved: *health domain workers*, *contact tracing operators*, and the *population*.

Public dissemination documents have been provided at the national level for *health domain workers* (including stakeholders).

The Istituto Superiore di Sanità, the Italian National Institute of Health, has proposed (and continues to propose) guidelines during the pandemic, on various issues related to the epidemic. These guidelines are called *Istituto Superiore di Sanità Covid Report* and they are all available in the Italian language [18]. Many of these reports are also available in the English language [19].

During the *start-up* period of the *Italian Digital Contact Tracing*, three reports [20,21,22], dedicated or strongly correlated to DCT were proposed. The last had two versions: the first one was in May 2020, and the last one in October 2020. These three reports [20,21,22] dealt with three aspects of the *health domain* that are closely related to DCT: the traditional CT [20], DCT [22], and the impact of ethics in DCT [21]. This is to inform, update, and raise awareness among workers in the *health domain*.

The *first report* [20] highlighted how contact tracing is a key component of COVID-19 prevention and control strategies. Furthermore, the report explained the aim of contact tracing to rapidly identify secondary cases and prevent further transmission of infection, and described the key phases of contact tracing in Italy.

The *second report* [21] highlighted that DCT raises multiple relevant ethical issues involving various areas: organization of health services, public health, clinical medicine, social medicine, epidemiology, technology, law, and many other areas. Furthermore, it reported some crucial elements from an ethical point of view, which included the evaluation of effectiveness, the separation of personal data from public health data, transparency, information, and the solidarity dimension (for example, helping the less capable with technologies) that must characterize any public health action.

The *third report* [22] had three perspectives. The first one introduced contact tracing, starting from the definition of the World Health Organization and independently from the digital techniques. The second point of view highlighted the innovations of mobile technology, based on smartphones connected to DCT. The third point of view dealt with the diffusion and evolution of these apps through an analysis of state-of-the-art technology.

The Istituto Superiore di Sanità coordinated online courses at a national level and proposed them to the contact-tracing operators [23]. Specific training was also provided on the app, “Immuni”. The remote training methods allowed both the enlargement of the prospective number of the trained subjects and maintained social distancing. *The general population also* received information on the app, “Immuni” through the mass media (the internet, radio, newspapers, and public posters).

## 4. Deployment and Current Use of the App, “Immuni”

The section analyzes the deployment and use of the app, also taking into consideration parameters relating to the digital divide, the estimates of truly positive subjects based on seroprevalence, and economic indicators. Table 1 reports the description of the topic considered, the source referring to it, and the relative indexed scientific references (web, report, and study) accessed at the date of writing the piece (5 October 2021). The acronyms used are also shown in the list of acronyms before the references. References are available in [24,25,26] (Table 1) and provide the numerical data related to: (a) the daily numerical downloads; (b) the daily number of *diagnosed positives* to the virus, who accepted data storage; and (c) the number of notifications. Based on this data, we observe that *16,167,210* downloads were carried out; *25,720* positive users registered voluntarily; and *111,791* notifications were sent. The manufacturer says that the detection is partial, as all notifications for iOS devices are detected and only a third of those sent by Android have the necessary technology available to safely detect them.

It is interesting to compare these data with the national population. The Italian population amounts to *59,257,566* [27] (Table 1); therefore, a fraction of *16,167,210/59,257,566 = 0.2728* of the Italian population downloaded the app (*27.28%*). The number of *diagnosed positive subjects* (*DPS*) since the start of the pandemic is *4,683,646* [28] (Table 1). The number of DPS is much lower [29,30] than the number of *really positive subjects* (RPS). The ability to diagnose positive subjects depends on many factors, ranging from medical knowledge and up to citizen participation and diagnostic power. It changes from nation to nation. In Italy, a national survey was conducted [30] to estimate the RPS. From 25 May to 15 July 2020, the seroprevalence investigation on SARS-CoV-2 was carried out in accordance with the provisions of the law decree 10 May 2020 n. 30 “Urgent measures in the field of epidemiological and statistical studies on SARS-CoV-2”, converted into law on 2 July 2020.

The latest updated data from the national survey conducted by the Italian Ministry of Health [29,30] (Table 1) estimated that the number of *RPS* is up to six times greater that *DPS*:

(1) RPS=6×DPS

Given that new and updated epidemiological investigations could lead to corrections of this value, we can parametrize this relationship.

(2) RPS=K×DPS

Considering that the study was conducted at the beginning of the pandemic, when diagnostic capabilities and resources were still limited, we can consider the value of *K* = 6 as the maximum value. We need also to consider the impact of the *Digital Divide* on the percentage of population, reported above 27.28%, who downloaded the app. We must count the individuals who do not own a smartphone and consider them. In Italy, according to the data of the national census, conducted shortly before the pandemic, 73.8% [31] (Table 1) of the population had a smartphone. In this case, the ratio between the app downloads and the population that own smartphones is 0.37. According to the data of the latest national census (available on 6 October 2021), this value had increased to 83.3% [32] (Table 1). In this second case, the ratio between the app downloads and the population that own smartphones is 0.33.

**Figure 1:**
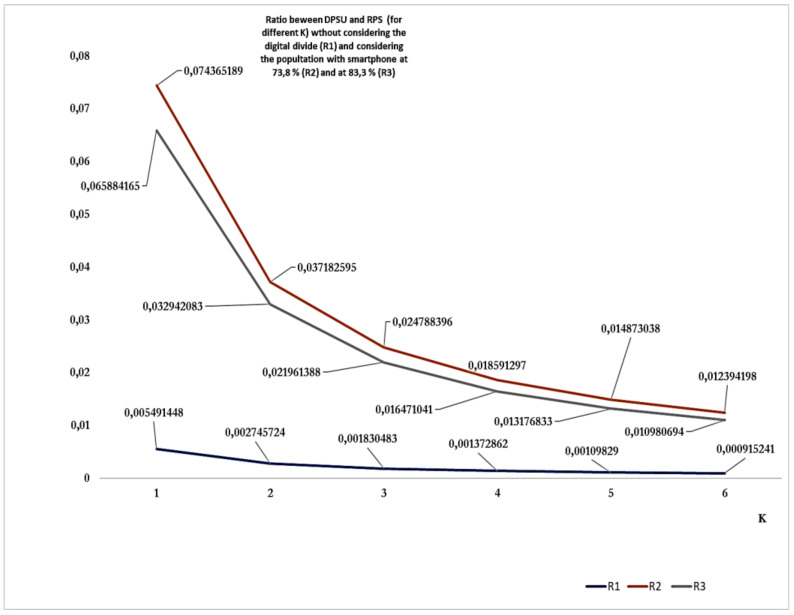
Ratio between the DPSU in the DCT system and the RPS (for different values of K): without the different impact of the digital divide (not considered, R1); considering the two different estimates of the digital divide at 73.8% (R2) and 83.3% (R3).

Figure 1 shows the ratio between the *diagnosed positive subjects uploaded* (DPSU) in the DCT system and the RPS for different values of K in three cases: (a) without considering the impact of the digital divide (not considered, R1). (b) Considering the two different estimates of the digital divide at 73.8% (R2) and 83.3% (R3). The best estimate considering the digital divide indicates a value never higher than 7.5 ‰, while the best estimate without considering the digital divide indicates a value never higher than 5.0 ‰.

**Figure 2:**
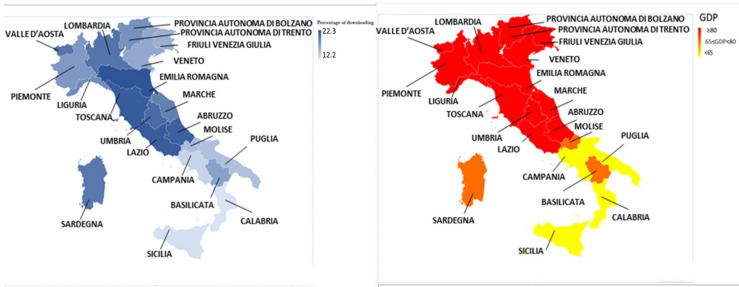
Graphic representation of percent of downloads for each region and GDP.

We can also identify the percent of downloads *(*%*D*) for each region [33,34] (Table 1). Table 2 shows these values for people with an age over 14 years. The region with the highest %*D* was Emilia Romagna, with 22.3%. The region with the lowest %*D* was Calabria, with 12.2%. An interesting result emerges if we consider the data relating to %*D* at a regional level compared to the *gross domestic product per capita (GDP)* [35,36] (Figure 2). Table 2 shows that: (a) the Italian regions with the largest *GDP* (≥80) all have a %*D* > 15%. (b) Regions with a lower *GDP* (<65) performed a %*D* <15%. (c) The regions with an intermediate *GDP* (65 ≤ GDP < 80 demonstrated a different behavior (*Molise* demonstrated %*D* <15%, *Sardegna and Basilicata* demonstrated %*D* > 15%).

## 5. Discussion and Conclusions

### 5.1. Our Contributions

The technological innovation of DCT has certainly characterized this pandemic compared to the previous ones. Considerable support was expected from the apps for DCT, based on the first studies [3]. Now, many months after the start of the use of this technology, scholars are wondering [37] what has been the real contribution of these apps to contact tracing and the fight against the pandemic. In our contribution, we first recalled the evolution of the technology, the development and diffusion that these apps have had worldwide, accompanied by a conspicuous and noteworthy increase in scientific output. Then we focused on the Italian DCT and retraced the introduction of the “Immuni” app. We highlighted that the introduction of this app was accompanied by awareness-raising initiatives for *health domain workers* and *contact-tracing operators* [20,21,22,23]. We have finally taken stock of the current deployment and uptake in Italy, noting underlying factors.

### 5.2. The Limits in the Deployment and the Current Use of DCT in Italy

Despite the initiatives undertaken, the deployment and the current use in Italy have shown limits. Only about a quarter of the population downloaded the app. A very low number of DPS (a fraction of the RPS, which was estimated to be even six times higher) uploaded their data. This number is around 7.5 ‰, if we consider the digital divide, and around 5.0 ‰ if we do not consider the digital divide. Among the factors that contributed to a higher/lower downloading, although not by much, we identified the digital divide and, at the regional level, the GDP, which accounts for several sub-factors (e.g., social factors, infrastructure, technology, education, and health).

### 5.3. The Impact of the Digital Divide

A notable part of the population certainly could not take advantage of these technologies due to the digital divide. The digital divide is a very key aspect and depends on two very important parameters: *literacy* [38] and access to *infrastructures* [39]. This value, with reference to the access to mobile technology in the period immediately preceding the pandemic, was equal to 26.2% [31] and then decreased to 16.7% [32]. We do not have information regarding the intention of this lost population group to join DCT. However, assuming a uniformity of behavior within the population, the contribution of this group would not have changed the conclusions.

Information on the social demographic influence on the digital divide is not directly available due to privacy. However, previous studies based on questionnaires reveal that some categories (e.g., elderly) were not familiar with the Italian DCT [40]. In Italy, important initiatives to minimize the digital divide were undertaken in this period, both in terms of *literacy* and *infrastructure*. National cashback programs on reimbursement with debit and credit cards, managed by an app, motivated the approach and familiarization with mobile technology [41]. The possibility of providing shopping vouchers [42] to individuals from low socio-economic groups, dedicated to the purchase of mobile devices and the internet, is an initiative that has improved access to *infrastructures*.

All of these initiatives have contributed to bridging the digital divide, thereby increasing the number of citizens with smartphones from 73.8% to 83.3%. However, as we have seen, this has not consistently improved the use of DCT.

### 5.4. Factors Influencing Adoption of the App Based on the Literature

The evidence that we report in the analysis is consistent with what is emerging in the recent reviews available from scientific literature. Our study has begun to highlight some factors that have influenced the distribution of the app. The scientific literature has highlighted how, in general, there are more *design factors*, connected to the technological choice, and more *transversal factors* concerning acceptability and desirability (Table 3 provides a summary). Of course, these factors are also interconnected (e.g., desirability is linked to design factors). As far as the *design factors* are concerned, we highlight how the “Immuni” app is an app based on *proximity tracing* with *a high level of privacy*, dedicated *almost exclusively to DCT.*

The scientific literature on these specific points connected to the *design factors* has produced clear evidence of:

- *The limits of the proximity tracing*

In general, we note [37] the limit of *proximity technologies*, using Bluetooth, in discovering cases of COVID-19. It has been underlined in [37] that the proximity detection using low energy Bluetooth was a small contribution to the detection of cases of COVID-19.

- *High levels of compliance with standards of data privacy are limiting*

Some studies have shown that the app, “Immuni”, is one of the apps with the greatest respect for privacy, with a very low amount of data collected [9]. Some studies confirm that apps with high levels of compliance with standards of data privacy (and “Immuni” is one of them) tend to fulfill public health interests to a limited extent and DCT with a lower level of data privacy protection allow for the collection of more data [43].

- *High level of integration of functions could improve the use*

We have seen how the integrations of greater functionality with DCT (including connection functions with social media) have been lacking in the apps developed for COVID-19 [12]. An expansion of the offer of functions could probably improve the use of the “Immuni” app. It should be noted that the app, “Immuni”, is already moving in this direction, allowing, for example, one to download the vaccination certificate.

There are many transversal factors that still need to be explored. It is important to focus on protocols for the clear identification of these factors. Furthermore, it is also important both to investigate the desirable requirements that an app for DCT must have and design bottom-up mechanisms to understand the failure factors. We have rephrased. In addition, on these aspects, the scientific literature is supporting us and could be extended to the Italian DCT experience:

- Some works are moving towards the definition of protocols for the correct identification of the factors [44].
- Some authors have focused on the desirable requirements that a DCT app must have to be successful and have made them explicit [14].
- Other authors went to the field to review the reports on the app stores relating to these apps [8] to understand what the users were not satisfied with.

### 5.5. Final Reflections and Further Work

A recent study available on the Cochrane database [45] traced both the reflections and the future directions and efforts in DCT. We strongly share this position based on the outcome from randomized controlled trials, cluster-randomized controlled trials, quasi-randomized controlled trials, cohort studies, cross-sectional studies, and modeling studies in general populations (all very important sources for evidence-based medicine).

The key takeaways from this review are as follows:

- There is very low-certainty evidence that DCT may produce more reliable counts of contacts and reduce time to complete contact tracing.
- Stronger primary research on the effectiveness of contact tracing technologies is needed.
- Future studies should better consider the access, acceptability, and equity.
- Studies should focus on the relationships between acceptability of DCT and the impact of the privacy that can hamper the diffusion of this technology.

We believe that a field survey could help us a lot to face the above-listed key takeaways and to focus on all the emerging issues to understand which factors have influence, what are the design suggestions of the population, and what is lacking in acceptability. Certainly, electronic questionnaires, designed for the population, could be useful [40], as they have already been used in the USA for many issues related to the pandemic [46]. Our idea is, because they have already been used, to continue this path by proposing a dedicated national questionnaire, also based on a *community engaged approach,* involving all the actors (*health domain workers*, *contact tracing operators*, and the general population) that for the apps, such as “Immuni”, could give useful feedbacks for the improvement of their use [47].

## Figures and Tables

**Table 1:** Summary table with the description of the data considered, the direct or indirect source, and the references (* accessed at the date of writing, 5 October 2021).

Description	Sources (Direct or Indirect)	Reference and Year
Statistics on people owning smartphones in Italy.	CENSIS (Italian national body designated for social research) reports	N. 31 (2019), N. 32 (2021)
Statistics on the use of the app “Immuni” (downloading, uploading of diagnosed positive subjects, etc.)	GitHub and app “Immuni” Webs	N. 15–17, N. 24–26, N. 33–34 (*)
Statistics on *gross domestic product* per capita (GDP)	Eurostat (European body designed for European statistics) reports	N. 35–36(Updated 3 march 2021)
Statistics on Italian population	ISTAT (Italian national body designated for social research) reports	N. 27 (*)
Serological investigation on COVID-19In Italy	ISTAT (Italian national body designated for social research) reports	N. 28–29 (2021)
Statistics on COVID-19 in Italy	Data from Italian Ministry of health	N. 28 (*)

**Table 2:** Tabular representation of percent of downloads for each region and GDP.

Region	Percent of Downloads for Each Region	GDP
Abruzzo	21.5	GDP ≥ 80
Basilicata	16.9	65 ≤ GDP < 80
Calabria	12.2	GDP < 65
Campania	13.3	GDP < 65
Emilia-Romagna	22.3	GDP ≥ 80
Friuli Venezia Giulia	15.8	GDP ≥ 80
Lazio	21.7	GDP ≥ 80
Liguria	18.3	GDP ≥ 80
Lombardia	20.1	GDP ≥ 80
Marche	19.2	GDP ≥ 80
Molise	14.9	65 ≤ GDP < 80
Piemonte	17.5	GDP ≥ 80
Puglia	14.6	GDP < 65
Sardegna	19.8	6 ≤GDP < 80
Sicilia	12.5	GDP < 65
Toscana	21.8	GDP ≥ 80
Provincia autonoma di Trento	19.4	GDP ≥ 80
Provincia autonoma di Bolzano	16.7	GDP ≥ 80
Umbria	20.7	GDP ≥ 80
Valle d’Aosta	20.0	GDP ≥ 80
Veneto	16.4	GDP ≥ 80

**Table 3:** Articles on DCT recalled with a brief description of their focus.

Ref	Cited Article	Brief Description of the Focus
[8]	Garousi V, Cutting D, Felderer M. Mining user reviews of COVID contact-tracing apps: An exploratory analysis of nine European apps. J Syst Softw. 2021	Authors went to the field to review the referees relating to these apps to understand what the users were not satisfied with.
[9]	Elkhodr M, Mubin O, Iftikhar Z, Masood M, Alsinglawi B, Shahid S, Alnajjar F. Technology, Privacy, and User Opin-ions of COVID-19 Mobile Apps for Contact Tracing: Systematic Search and Content Analysis. J Med Internet Res. 2021 Feb 9;23(2):e23467. doi: 10.2196/23467. PMID: 33493125; PMCID: PMC7879719 Nov 4:111136. doi: 10.1016/j.jss.2021.111136. Epub ahead of print. PMID:34751198; PMCID: PMC8566091	Reviewed different apps for DCT, highlighted that *the app, “Immuni”, is one of the apps with the greatest respect for privacy, with a very low amount of data collected.*
[12]	Alanzi T. A Review of Mobile Applications Available in the App and Google Play Stores Used During the COVID-19 Outbreak. J Multidiscip Healthc. 2021 Jan 12;14:45–57. doi: 10.2147/JMDH.S285014. PMID: 33469298; PMCID: PMC7812813	Highlighted that a large integration of functionalities are lacking in the apps developed for the COVID-19.
[13]	Kahnbach L, Lehr D, Brandenburger J, Mallwitz T, Jent S, Hannibal S, Funk B, Janneck M. Quality and Adoption of COVID-19 Tracing Apps and Recommendations for Development: Systematic Interdisciplinary Review of European Apps. J Med Internet Res. 2021 Jun 2;23(6):e27989. doi: 10.2196/27989. PMID: 33890867; PMCID: PMC8174558	The study faced the quality in the apps for DCT. It used the mobile app rating scale to assess the app quality.
[14]	O’Connell J, Abbas M, Beecham S, Buckley J, Chochlov M, Fitzgerald B, Glynn L, Johnson K, Laffey J, McNicholas B, Nuseibeh B, O’Callaghan M, O’Keeffe I, Razzaq A, Rekanar K, Richardson I, Simpkin A, Storni C, Tsvyatkova D, Walsh J, Welsh T, O’Keeffe D. Best Practice Guidance for Digital Contact Tracing Apps: A Cross-disciplinary Review of the Literature. JMIR Mhealth Uhealth. 2021 Jun 7;9(6):e27753. doi: 10.2196/27753. PMID: 34003764; PMCID: PMC8189288	Authors reviewed the desiderable requirements that a DCT app must have to be successful and have made them explicit.
[37]	Maccari L, Cagno V. Do we need a contact tracing app? Comput Commun. 2021 Jan 15;166:9–18. doi: 10.1016/j.comcom.2020.11.007. Epub 2020 Nov 19. PMID:33235399; PMCID: PMC7676320	It has been underlined that the proximity detection using BLTE gave a low contribute to the detection of cases.
[43]	.Kolasa K, Mazzi F, Leszczuk-Czubkowska E, Zrubka Z, Péntek M. State of the Art in Adoption of Contact Tracing Apps and Recommendations Regarding Privacy Protection and Public Health: Systematic Review. JMIR Mhealth Uhealth. 2021 Jun 10;9(6):e23250. doi: 10.2196/23250. PMID: 34033581; PMCID: PMC8195202	Showed that apps with high levels of compliance with standards of data privacy (and “Immuni” is one of them) tend to fulfill public health interests to a limited extent and DCT with a lower level of data privacy protection allow for the collection of more data.
[44]	Oyibo K, Sahu KS, Oetomo A, Morita PP. Factors Influencing the Adoption of Contact Tracing Applications: Protocol for a Systematic Review. JMIR Res Protoc. 2021 Jun 1;10(6):e28961. doi: 10.2196/28961. PMID: 33974551; PMCID: PMC8171387	The study proposed protocols for the correct identification of the factors influencing DCT.
[45]	Anglemyer A, Moore TH, Parker L, Chambers T, Grady A, Chiu K, Parry M, Wilczynska M, Flemyng E, Bero L. Digital contact tracing technologies in epidemics: a rapid review. Cochrane Database Syst Rev. 2020 Aug 18;8(8):CD013699. doi: 10.1002/14651858.CD013699. PMID: 33502000; PMCID:PMC8241885	The study on the Cochrane database system review traced both the reflections and the future directions and efforts in DCT. The outcome from randomized controlled trials (RCTs), cluster-RCTs, quasi-RCTs, cohort studies, cross-sectional studies, and modeling studies in general populations was considered.

## Data Availability

Data sharing not applicable.

## Abbreviations

CT	Contact tracing
DCT	Digital contact tracing
RPS	Really positive subjects
DPS	Diagnosed positive subjects
DPSU	Diagnosed positive subjects uploaded
%D	Percent of downloads
GDP	Gross domestic product per capita
